# Evolutionary medicine Carpal tunnel syndrome

**DOI:** 10.1093/emph/eox006

**Published:** 2017-03-01

**Authors:** Althea Anne D. Perez, Scott W. Simpson

**Affiliations:** 1 Department of Biology, Case Western Reserve University, Cleveland, OH 44107, USA; 2 Department of Anatomy, School of Medicine, Case Western Reserve University, Cleveland, OH 44106, USA

## CARPAL TUNNEL SYNDROME

Carpal tunnel syndrome (CTS) results from median nerve entrapment during increased intra-tunnel pressure that is often a product of swelling of the digital flexor tendon sheaths consequent to repetitive movements of the hand and wrist or by direct compression on the wrist. Individuals with CTS may experience pain, tingling, numbness and weakness in the palmar surface of the first three digits, small thumb and lateral two lumbrical muscles [1]. Occupational factors may exacerbate CTS, most notably, repetitive and forceful activities using the hand. Unfortunately, individuals affected with CTS may be unable to perform basic functions and cannot work. In 2014, the median number of days taken from work for CTS (32 days) was much greater than the average number of days taken off for other non-fatal illnesses (9 days) [2].

Measurements of the cross sectional area (CSA) of the proximal tunnel are effective in predicting CTS. In individuals presenting with CTS, the CSA of the median nerve is significantly larger than in unaffected individuals. Further, the nerve tunnel index (CSA median nerve/CSA tunnel) in affected individuals is much higher than in non-affected individuals. [3].

## EVOLUTIONARY PERSPECTIVES

**Figure 1.**
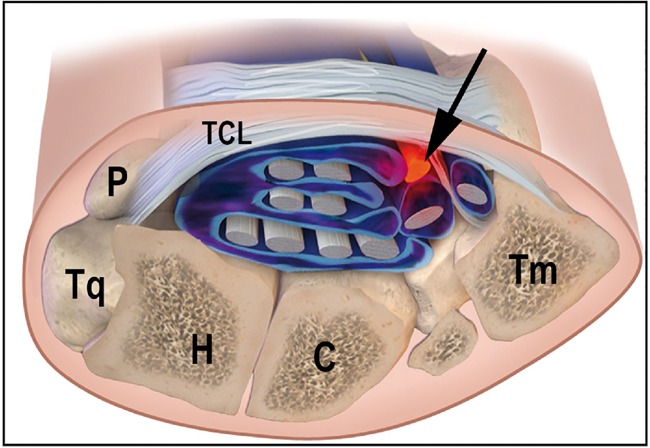
Cross section of a right proximal wrist, palmar side up. Arrow points to the median nerve. P, Pisiform; Tq, Triquetral; H, Hamate; C, Capitate; Tm, Trapezium; TCL, Transverse Carpal Ligament

CTS may arise exclusively in humans as a consequence of adopting bipedalism; the loss of forelimb usage during locomotion may have resulted in anatomical changes in the hand, including a reduction in the size and functional orientation of the pisiform bone. The proximal transverse carpal ligament attaches to the pisiform bone, forming the ‘roof’ of the carpal tunnel. The elongated, rod-shaped pisiform bone in apes serves as the medio-proximal border of the CT and increases the CSA (Fig. 1) [4, 5]. Increased carpal tunnel CSA increases the amount of space available for the median nerve and various other tendons and bursae that occupy the tunnel. Increased CSA would decrease the nerve tunnel index, which also may reflect a decrease the risk of CTS development [3].

**Figure 2.**
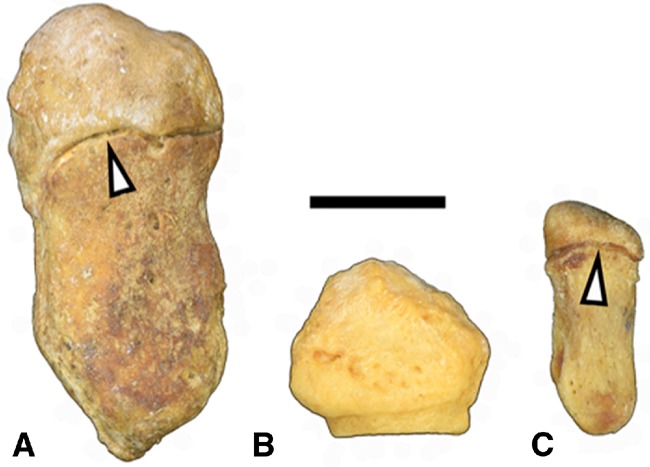
Pisiform bones. **(A)** Gorilla, **(B)** Human, **(C)** Chimpanzee pisiform bones. Arrows indicate secondary ossification centers. Scale bar 10 mm

Comparison of the pisiform bone length and development between humans and primates may shed light on the increased risk of CTS in humans. Unlike humans, apes retain a growth plate and secondary center of ossification in the pisiform, resulting in long pisiform bones (Fig. 2) that function as a lever to increase torque during wrist flexion while climbing or terrestrial knuckle-walking [5].

Common treatments for CTS include immobilization, corticoid injections, and surgery. Surgical incision of the flexor retinaculum is one of a few treatments known to permanently alleviate symptoms. Thus, there is a desire to explore more favorable strategies to alleviate CTS pathology.

Further research is needed to trace the exact function by which the biomechanical lever of the pisiform serves to increase the CSA of the carpal tunnel in non-human primates. Additional allometric analysis of the articular surface of the triquetrum in proportion to body size in other primate species would be necessary to better understand the anatomical factors that can increase risk in humans.
